# Bioinformatic analysis of WxL domain proteins

**DOI:** 10.1016/j.sjbs.2022.103526

**Published:** 2022-12-07

**Authors:** Mahreen U. Hassan, Mike P. Williamson

**Affiliations:** a School of Biosciences, The University of Sheffield, Western Bank, Sheffield S10 2TN, UK; b Dept of Microbiology, Shaheed Benazir Bhutto Women University, Peshawar 2500, Pakistan^1^

**Keywords:** WxL, Peptidoglycan, Virulence, Bioinformatics

## Abstract

The WxL domain is found on the cell surface of many bacteria, most of which are commensal gut bacteria. Its functions are generally identified as being related to virulence and/or peptidoglycan attachment, but there is so far no clear function or structure for this domain. Here, a range of bioinformatics tools were used to clarify the structure and function. These indicate that WxL domains occur in cell surface-associated gene clusters that always contain a small WxL, large WxL and DUF916 domain; and that the small and large WxL proteins have distinct structure despite sharing two conserved WxL motifs. The two WxL motifs form a hydrophobic surface buried inside the protein. The likely function of the WxL domain is to attach to bacterial peptidoglycan, forming a platform to allow associated domains in the cluster to interact with host proteins.

## 1 Introduction

The WxL domain contains 160 to 190 amino acids, and is characterized by two conserved motifs containing the sequence Trp-X-Leu (Galloway-Peña et al., 2015). It has been found in the genomes of low G-C gram-positive bacteria such as *Listeria monocytogenes*, *Enterococcus faecium* and *Enterococcus faecalis*. These species are typically commensal gut bacteria, which cause opportunistic infections in immunocompromised individuals and are often nosocomial. WxL proteins are typically found in gene clusters, named Csc (Cell surface cluster) in *Listeria* (Bierne and Cossart, 2007) and *Lactobacillus plantarum* (Siezen et al., 2006). The clusters contain a conserved DUF916 domain together with two different proteins that contain WxL sequences, which have been described as large and small WxL proteins (Galloway-Peña et al., 2015), on the basis of the length of the protein sequence, and in recognition of the observation that small WxL proteins contain only a signal sequence and WxL domain, while large WxL proteins contain additional domains between the signal sequence and WxL domain.

The gene clusters also often contain a protein with the LPxTG sequence motif, which is a motif that is recognised by the enzyme sortase and used to covalently attach the protein to peptidoglycan at the cell surface (Navarre and Schneewind, 1994). For this reason, and because of the presence of signal peptides at the *N*-terminal ends of proteins in the cluster, it is believed that the proteins in the cluster are exposed on the cell surface. The function of the gene cluster is not clear. It recognizes peptidoglycan (Brinster et al., 2007a), and may also have a role in virulence (Castro et al., 2017, Jamet et al., 2017, Nunez et al., 2018), possibly linked to a leucine-rich repeat domain found in some large WxL sequences (Brinster et al., 2007b). The role in virulence is supported by identification of WxL proteins in pathogenic strains of *E. faecalis* (Bourgogne et al., 2008, Solheim et al., 2011). It has also been suggested to have a role in digestion and utilization of polysaccharides by *L. plantarum* (Erkmen and Bozoglu, 2016, Siezen et al., 2006).

The aim of this study was to identify the roles of WxL domains. A range of bioinfomatics tools were used to investigate species distribution, cluster composition, and domain composition and structure. It is shown that that the core gene cluster contains a DUF916, small WxL and large WxL; and that although small and large WxL contain two conserved WxL sequences they have different structures. Predicted structures and interactions are presented for the WxL domains, which are shown to be β-sheet proteins. The WxL domains are likely to function as peptidoglycan-binding domains, forming a platform that permits interaction of other domains within the cluster with the host.

## 2 Materials and methods

### 2.1 Protein selections and sequence retrieval

Sequence retrieval was done by the help of Uniprot accession number (Supplementary Table S1). The distribution of WxL proteins was found through the Pfam data base https://pfam.xfam.org (Mistry et al., 2021).

### 2.2 Protein characteristics

Physicochemical properties were determined with the ProtParam tool available from ExPASy https://web.expasy.org/protparam. Protein location, signal peptides, and transmembrane helices were analysed using CELLO (https://cello.life.nctu.edu.tw/), TargetP 2.0 (https://www.cbs.dtu.dk/services/TargetP/), cNLS Mapper (https://nls-mapper.iab.keio.ac.jp/cgi-bin/NLS_Mapper_form.cgi), TMHMM server v. 2.0 (https://services.healthtech.dtu.dk/service.php?TMHMM-2.0), HMMTOP (https://www.enzim.hu/hmmtop/html/submit.html) and Protter (https://wlab.ethz.ch/protter/start/). Interaction networks were identified using the STRING database (https://string-db.org) with a score cut-off value of 0.40.

### 2.3 Domain and fold analysis

Domains, motifs and families were identified using InterProScan (https://www.ebi.ac.uk/interpro/result/InterProScan/), Conserved Domain Architecture Retrieval Tool (CDART) (https://www.ncbi.nlm.nih.gov/Structure/lexington/lexington.cgi),

Simple Modular Architecture Research Tool (SMART) https://smart.embl-heidelberg.de/smart/show_motifs.pl, BlastP https://blast.ncbi.nlm.nih.gov/Blast.cg, Motif finder https://www.genome.jp/tools/motif/, and the PFP-FunDSeqE predictor web server. InterProScan allows the scanning of sequences against the InterPro signatures collected from different databases (Jones et al., 2014). CDART searches protein similarities against the NCBI Entrez Protein Database using profiles of protein domains and scores them based on the domain architecture (Geer et al., 2002). SMART is a resource of manually curated protein domain models, which identifies, annotates and explores the architecture of protein domains (Letunic et al., 2015). Sequence alignments were done using MUSCLE (Multiple Sequence Comparison by log Expectation) https://www.ebi.ac.uk/Tools/msa/muscle/ (Edgar, 2004).

### 2.4 Structure prediction

Secondary structure was predicted using PSIPRED (McGuffin et al., 2000). Tertiary structure was predicted using AlphaFold https://alphafold.ebi.ac.uk (Jumper et al., 2021), Robetta https://robetta.bakerlab.org/ (Kim et al., 2004) and Phyre2 https://www.sbg.bio.ic.ac.uk/phyre2/ (Kelley et al., 2015). Protein topology was determined using PDBsum https://www.ebi.ac.uk/thornton-srv/databases/pdbsum (Laskowski et al., 2018). The structures produced were analysed in DALI (Holm, 2020) to identify related structures. Predictions were analysed using ProCheck, ERRAT, ProSA (Protein Structure Analysis), and Verify3D. The binding pockets were explored by 3D-Ligand and CastP.

## 3 Results

**Fig. 1:**
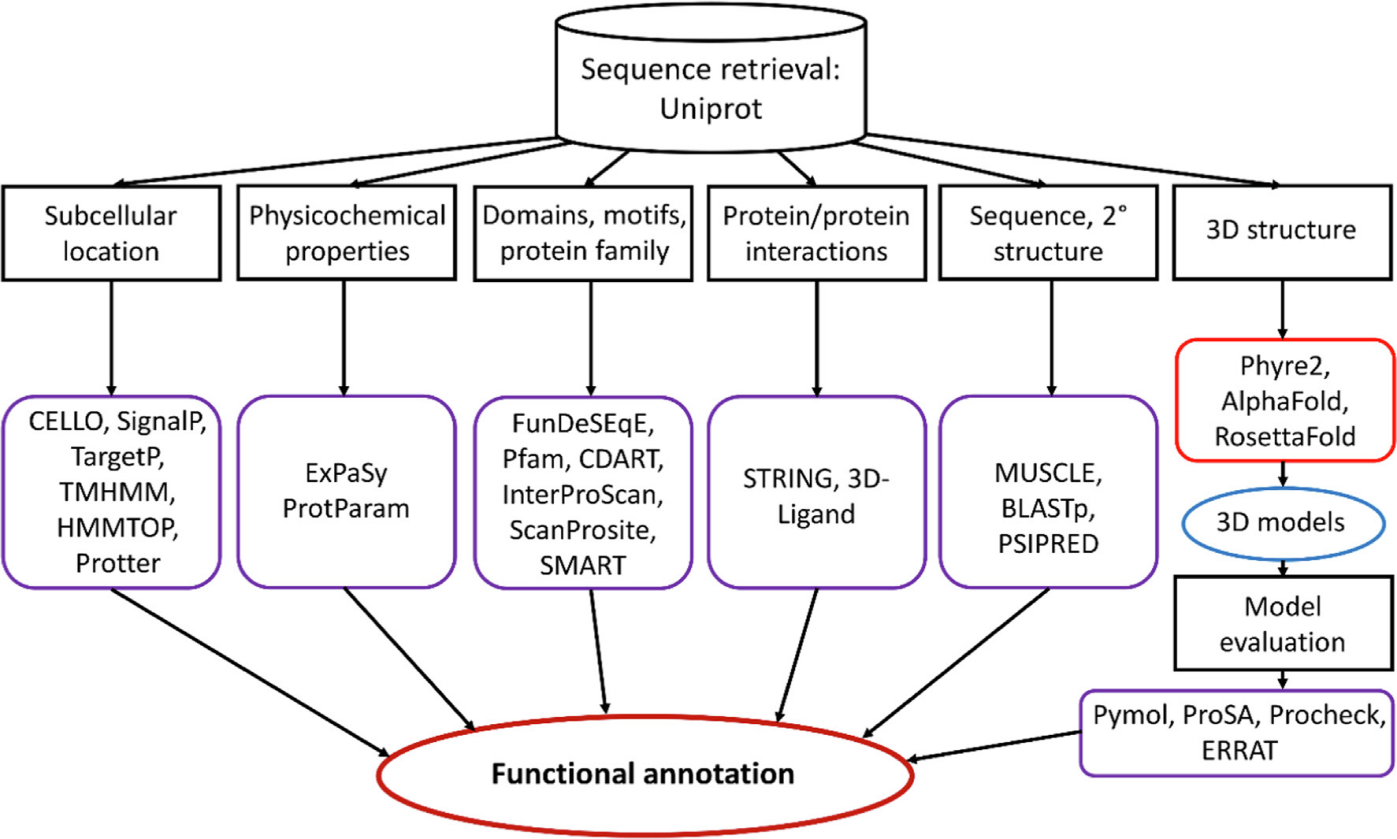
Flow chart of the methodology used here.

The properties and function of WxL proteins were examined using a suite of tools, summarised in Fig. 1.

### 3.1 The WxL sequence motif

**Fig. 2:**
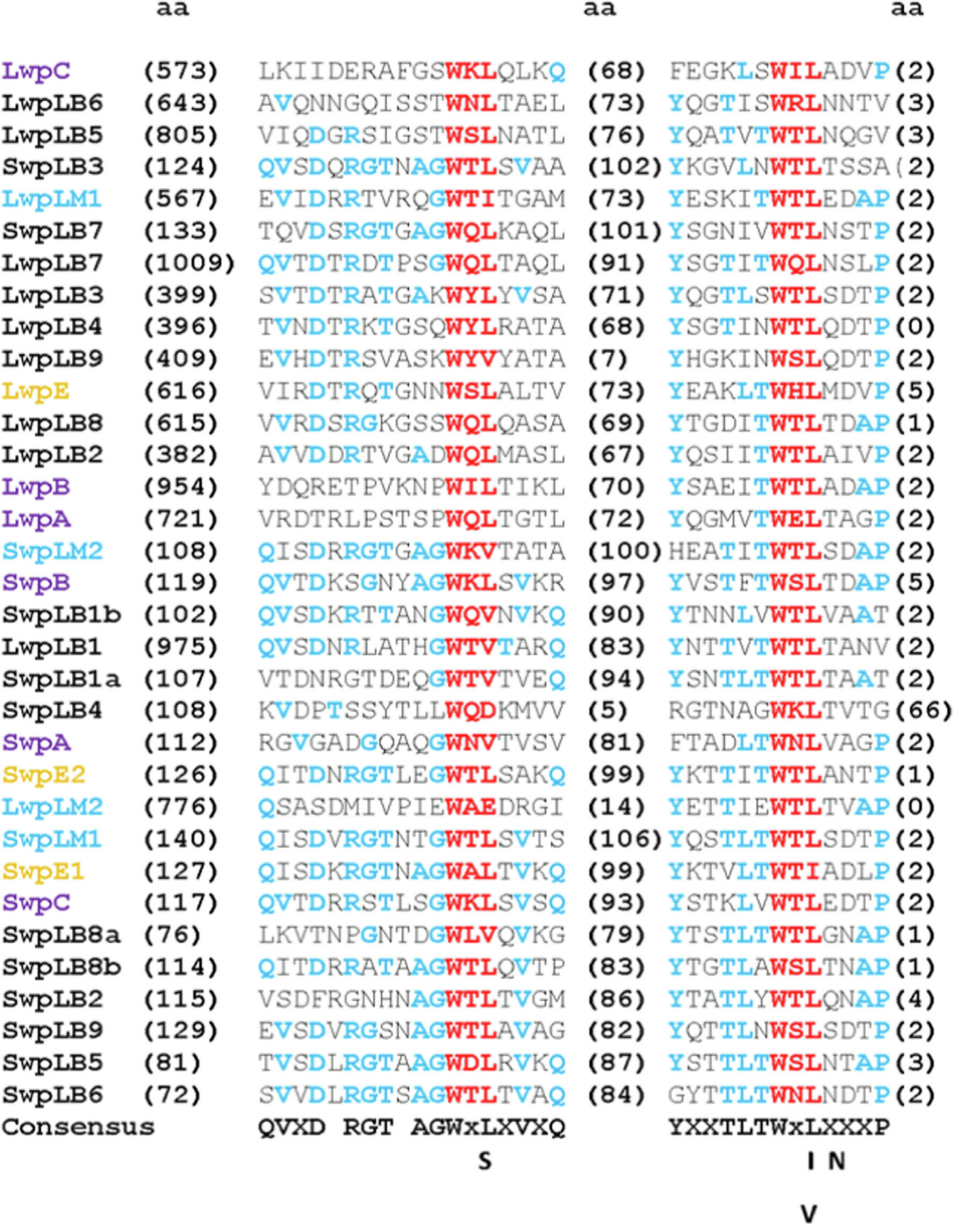
Multiple sequence alignment of 33 WxL proteins of different species using MUSCLE. The WxL sequences are in red; conserved amino acids are in blue, and summarised by the consensus sequence at the bottom. The numbers in parentheses denote the number of amino acids preceding, intervening and following these sequences in each protein. Lwp denotes long WxL protein, and Swp short WxL protein. Protein names in black are from *L. plantarum*; names in cyan from *L. monocytogenes*; names in purple from *E. faecium*, and names in brown from *E. faecalis*.

33 WxL protein sequences were compared using the MUSCLE server (https://www.ebi.ac.uk/Tools/msa/muscle/) (Edgar, 2004) and are shown in Fig. 2. All WxL proteins contain two well-conserved but different sequences, each with a WxL sequence motif. In the second motif, the amino acid x is small and hydrophilic, while in the first motif it is more variable. The amino acids surrounding the motif are conserved over a stretch of 13 residues in the second motif, and 18 in the first. They are conserved to roughly the same extent across both small and large WxL sequences. There are however clear differences between small and large WxL proteins: notably that small WxL consistently has about 110 residues preceding the first motif, while for large WxL this number is more variable but much larger. There is also a difference in the number of residues that separate the two motifs: roughly 68 for large and 106 for small WxL. For both large and small WxL proteins, the WxL domain always occurs at the C-terminal end of the sequence. These characteristics provide a readily identifiable profile for WxL domains, which provides a clear distinction between small and large proteins.

### 3.2 Species distribution of the WxL domain

**Fig. 3:**
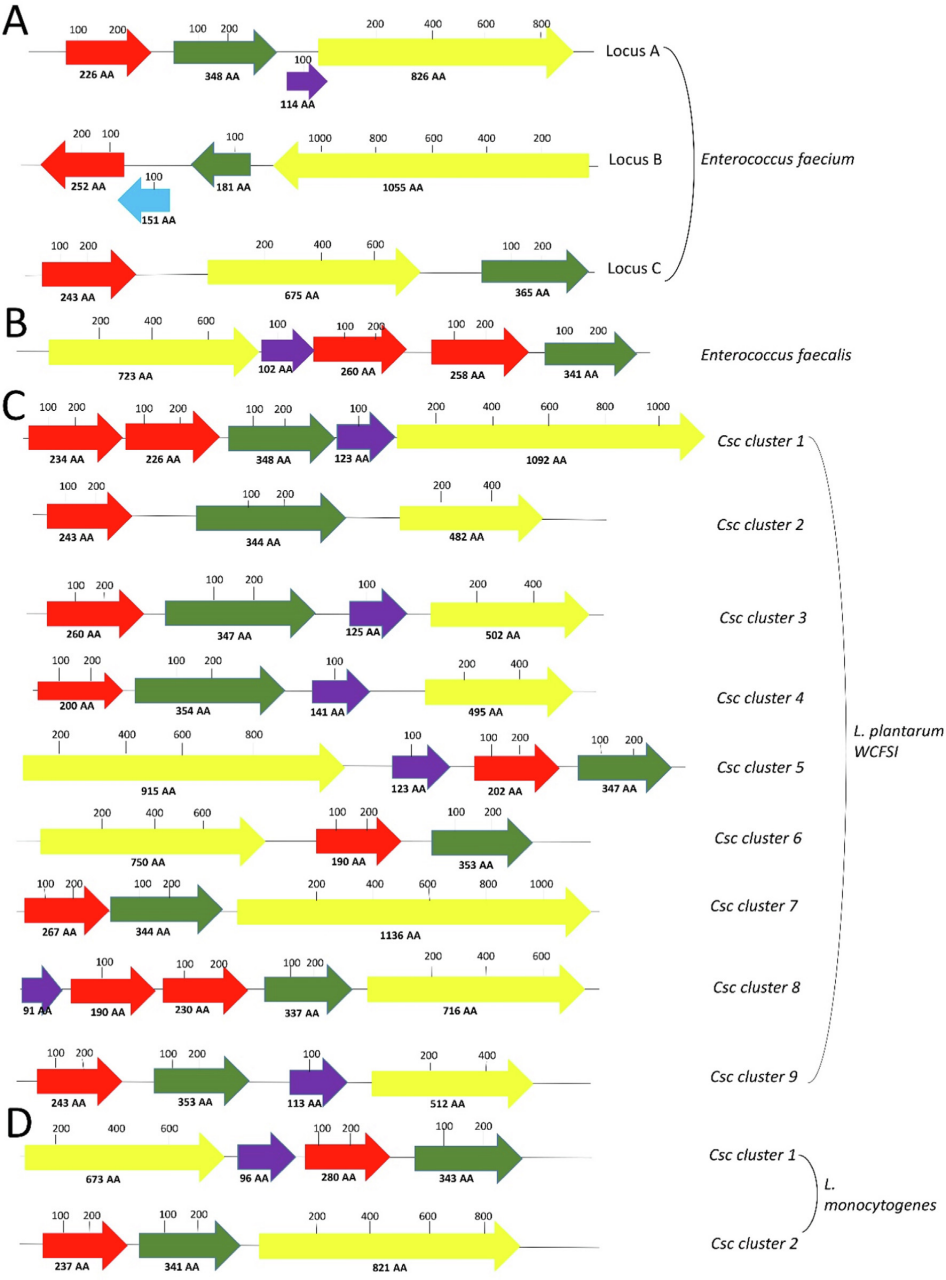
Schematic representation of genetic loci encoding WxL domain proteins in representative different species. The horizontal spacing is proportional to amino acid sequence length. Different domain types are colored differently: red, small WxL; green, DUF916; yellow, large WxL; purple, LPxTG protein.

WxL domains are exclusively found in bacteria (Fig. 3). According to Pfam release 33.1, there are 137 species potentially containing WxL domain proteins (El-Gebali et al., 2019). According to the current classification, there about 9,300 recognized species of prokaryotes including bacteria and archea (Louca et al., 2019). The species distribution of WxL domain proteins is therefore very limited and is mainly gut commensal species.

Over 91 % of the 938 WxL sequences known in bacteria are found in Firmicutes, of which 99 % are in Bacilli. The WxL motif is widely distributed in two Bacilli orders: Lactobacillales (755) and Bacillalles (96) (Supplementary Fig. S1).

### 3.3 Analysis of WxL gene clusters

As noted by previous authors (Brinster et al., 2007a, Galloway-Peña et al., 2015, Nunez et al., 2018, Siezen et al., 2006), WxL proteins occur in gene clusters, shown in Fig. 3. All these sequences have signal peptides at the *N*-terminus and are therefore secreted proteins.

The number, position and orientation of small and large WxL domains is not identical in all the species. Some species have one small WxL and one large WxL, while others have two small and one large WxL. It is of interest that all clusters contain a large WxL, a small WxL and a DUF916 domain, which therefore appears to be the minimal domain organisation. DUF916 is a poorly characterised Domain of Unknown Function.

The annotation shown in Fig. 3 differs slightly from published annotations. Siezen et al. (2006) annotated predicted proteins Ip1446 and Ip3412 as CscB (equivalent to small WxL) on the basis of their predicted pI, but the protein size, and the presence of additional predicted folded domains, clearly marks them out as large WxL. For similar reasons, ElrA of *E. faecalis* V583 (Nunez et al., 2018) is here annotated as a large WxL, and ElrC and ElrD as small WxL.

Most of the gene clusters also contain a LPxTG domain. This sequence motif is recognized by sortases and used to covalently attach the domain to peptidoglycan. This would thus appear to be a useful but not essential feature, and confirms the assumption that the proteins from the gene cluster are located on the outer surface of the peptidoglycan layer.

On the basis of these observations, and the species distribution noted above, we hypothesise that some of the proteins in the cluster attach the cluster to the peptidoglycan surface, and function to orient the remaining proteins in the cluster so that they point away from the bacterial surface and interact with the host. This would explain the observations noted in the Introduction, that the WxL cluster has a potential role in virulence.

### 3.4 Analysis of domain structure

The domains within the clusters have been further analysed. Small WxL proteins are predicted to consist of a single domain of around 180 residues, following on closely after the *N*-terminal signal sequence (Fig. S2; Table S2). They are generally predicted to have an acidic pI. The DUF916 protein is in most cases predicted to contain a DUF916 domain followed by a DUF3324 domain, usually with no other identifiable domains present in the protein. Neither of these domains has an experimentally determined structure or an assigned function. However, structure prediction programmes such as AlphaFold (discussed below) generate confident predictions that they adopt a β-sandwich structure, reminiscent of IgG or Fibronectin Type III domains.

**Fig. 4:**
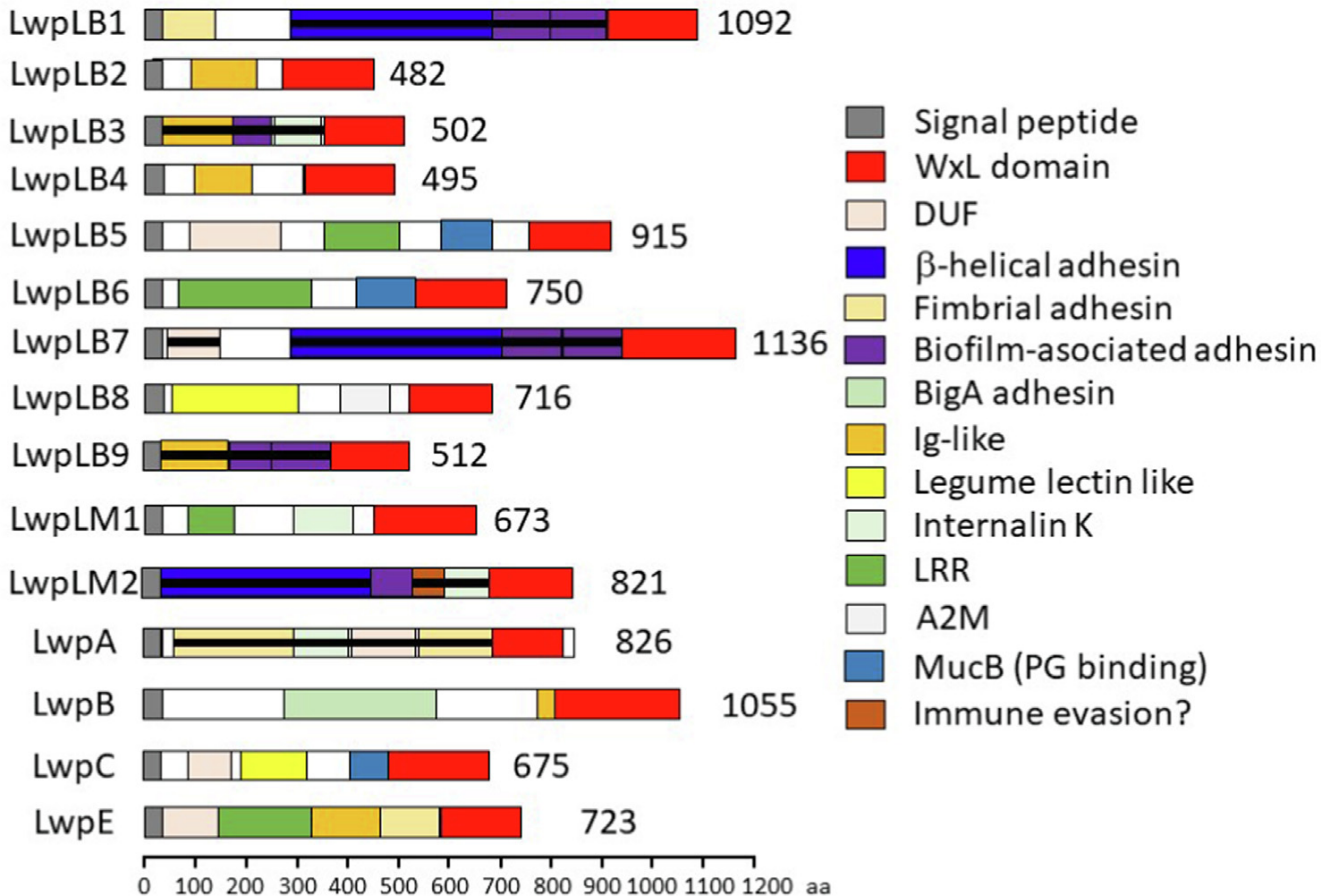
Schematic representation of Large WxL proteins. LwpLB1 through LwpLB9 are large WxL of *L. plantarum* WCFS1 from clusters 1 to 9; LwpM1/2 are large WxL of *L. monocytogenes* cluster 1/2; LwpA/B/C are large WxL of *E. faecium* DO locus A/B/C; and LwpE is the large WxL of *E. faecalis* V583. Domains with a black horizontal bar were identified using AlphaFold and RosettaFold.

The most interesting protein is the large WxL protein. The genes are much longer than those of the small WxL, with predicted proteins approximately 500–1000 residues long (Fig. 4). A motif analysis was conducted using a range of programs that analyse protein sequence, which succeeded in identifying many of the domains present (Table S3), but left large sections of some genes unidentified, corresponding to the domains with a black horizontal bar in Fig. 4. In order to obtain further information on these proteins, AlphaFold, Robetta and RosettaFold were run. They generated similar predictions in all cases (Table S4). The three-dimensional predictions were then analysed using DALI to identify possible functions.

**Fig. 5:**
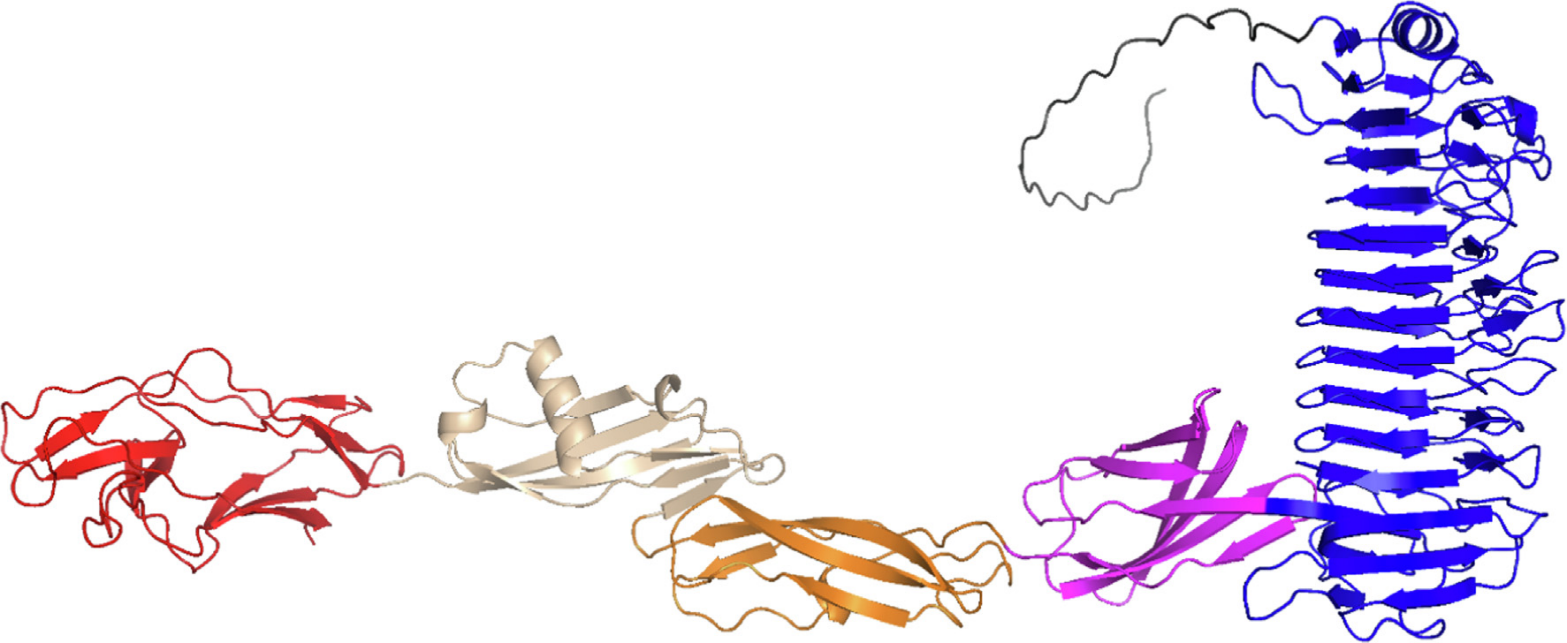
Structure for LwpLM2 large WxL predicted by AlphaFold. The domains are shown using the same color scheme as Fig. 4. The *N*-terminus is at the right. In order from the *N*-terminus, there is a signal peptide (grey) (which is presumably absent in the mature protein), a β-helical adhesin domain (blue), a biofilm-associated adhesin domain (magenta), a domain similar to the Streptococcal R4 surface protein that may have a role in immune evasion (brown), an internalin K domain (salmon), and a large WxL domain (red). Note the linear arrangement from the WxL outwards.

All of the large WxL proteins are predicted to contain a series of folded domains, these being generally almost continuous. It is striking that the WxL domain is always the C-terminal domain. Most of the other domains are largely antiparallel β-sheet proteins, with a general resemblance to the IgG or Fibronectin III domain, with the striking exception of a β-helical adhesin domain (indicated in navy blue in Fig. 4) and the structurally related β-helical leucine-rich repeat (LRR), which are found in a few of the clusters. Many of the domains identified are likely to have a role in adhesion, specifically to a eukaryotic host. These include the β-helical adhesin, fimbrial adhesin (with similarities to domains at the tip of bacterial fimbria or pili), biofilm-associated adhesin, BigA adhesin, Ig-like, internalin, LRR and A2M domains. Given that the WxL domain is always C-terminal, and that this is therefore the last domain to be expressed and secreted, the clear conclusion is that the WxL domain functions to anchor the large WxL to the bacterial cell wall, and present the other domains to interact with the host. In support of this proposal, we note that the structural predictions of AlphaFold and RosettaFold almost always have the domains extending in a linear arrangement out from the WxL domain, as illustrated by the AlphaFold prediction for LwpLM2, shown in Fig. 5.

It is worth adding that it has been proposed (Brinster et al., 2007a) that in *E. faecalis* the C-terminal WxL domain (named ElrA in that work) is preceded by a FHL2-interacting domain (FID), starting at residue 607. Analysis using RosettaFold and AlphaFold shows that in fact the domain boundary is at residue 581, with the FID domain being a fimbrial adhesin homologue.

### 3.5 Structure analysis of WxL domains

Previous analyses of WxL domains were unable to model a structure (Galloway-Peña et al., 2015). Galloway-Peña et al. (2015) subsequently ran DisEMBL and concluded that these proteins are highly disordered proteins. However, the tools available are now better. Here, Phyre2, Robetta, RosettaFold and AlphaFold were used to predict structures for WxL domains. They all predict similar structures, with similar and reasonably good degrees of success (Tables S5-S8).

**Fig. 6:**
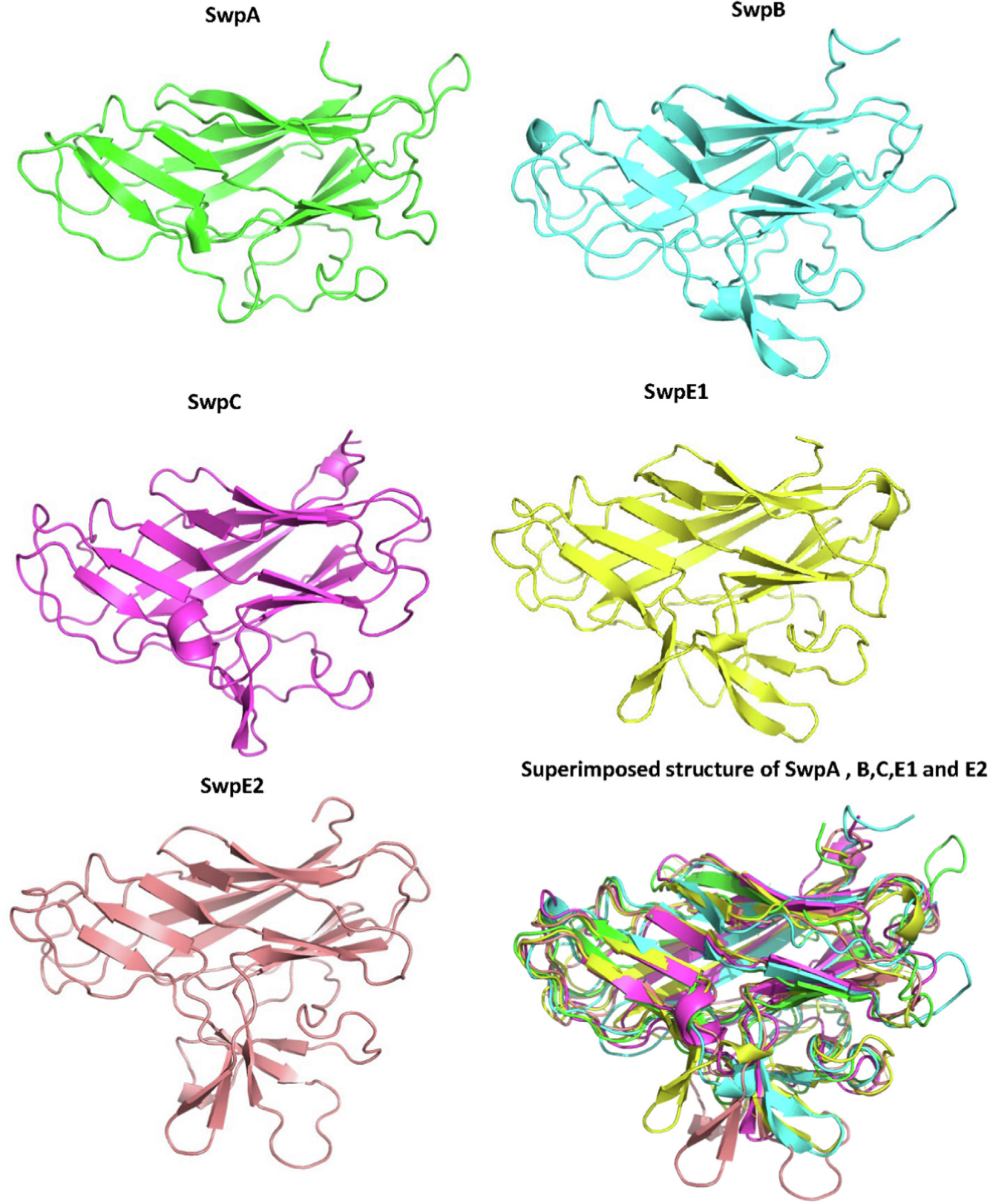
AlphaFold predictions for the small WxL domains SwpA, B, C, E1, and E2. The predicted structures are very similar and superimpose well.

**Fig. 7:**
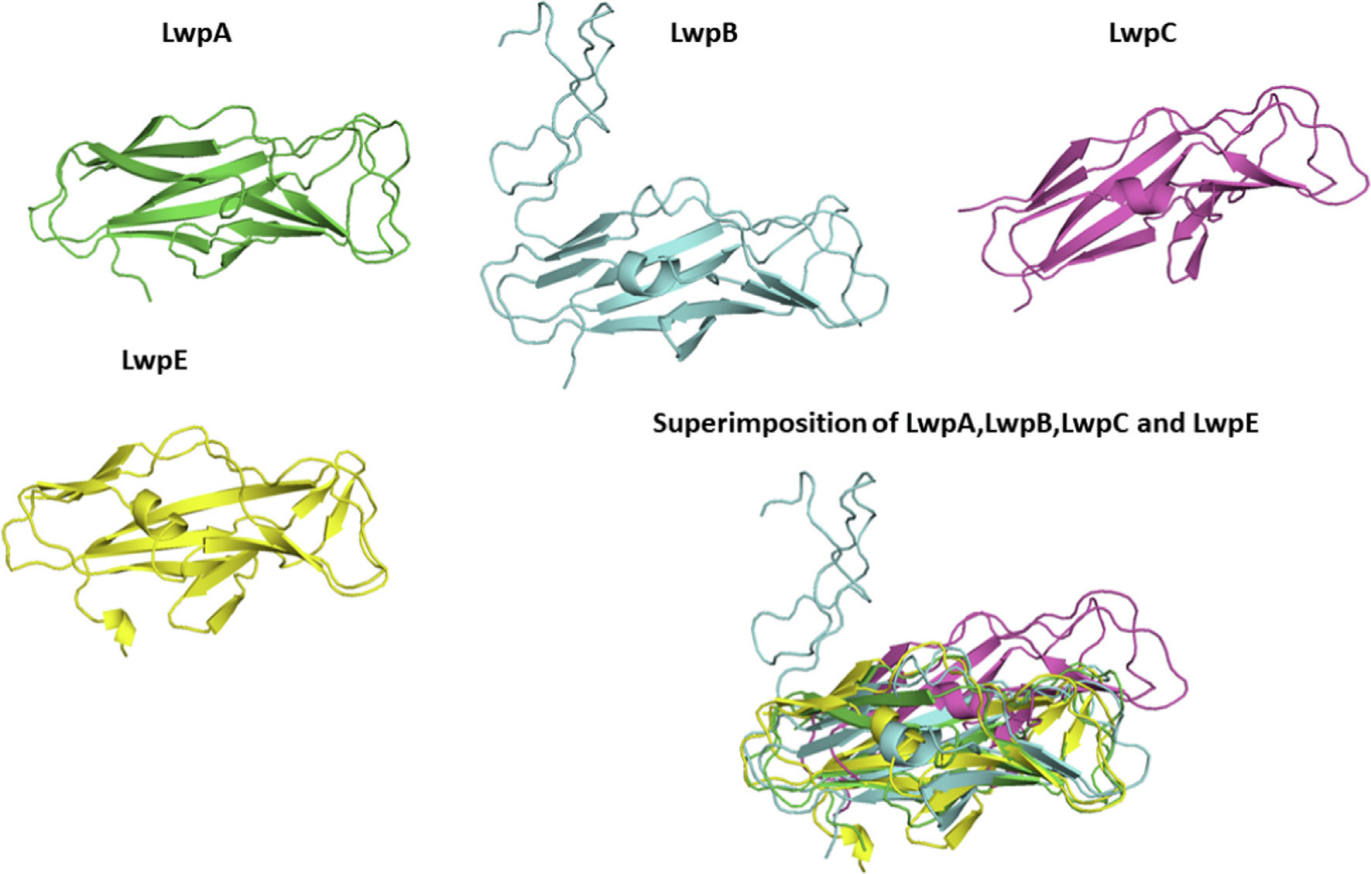
AlphaFold predictions for the large WxL domains from LwpA, LwpB, LwpC and LwpE. The structures agree well with each other. Structure predictions by Robetta are similar though slightly less complete (Fig. S3).

The models for small WxL domains are highly superimposable (Fig. 6), providing a high degree of confidence that the model is reliable. Similarly, the models for the large WxL domain also agree well (Fig. 7).

**Fig. 8:**
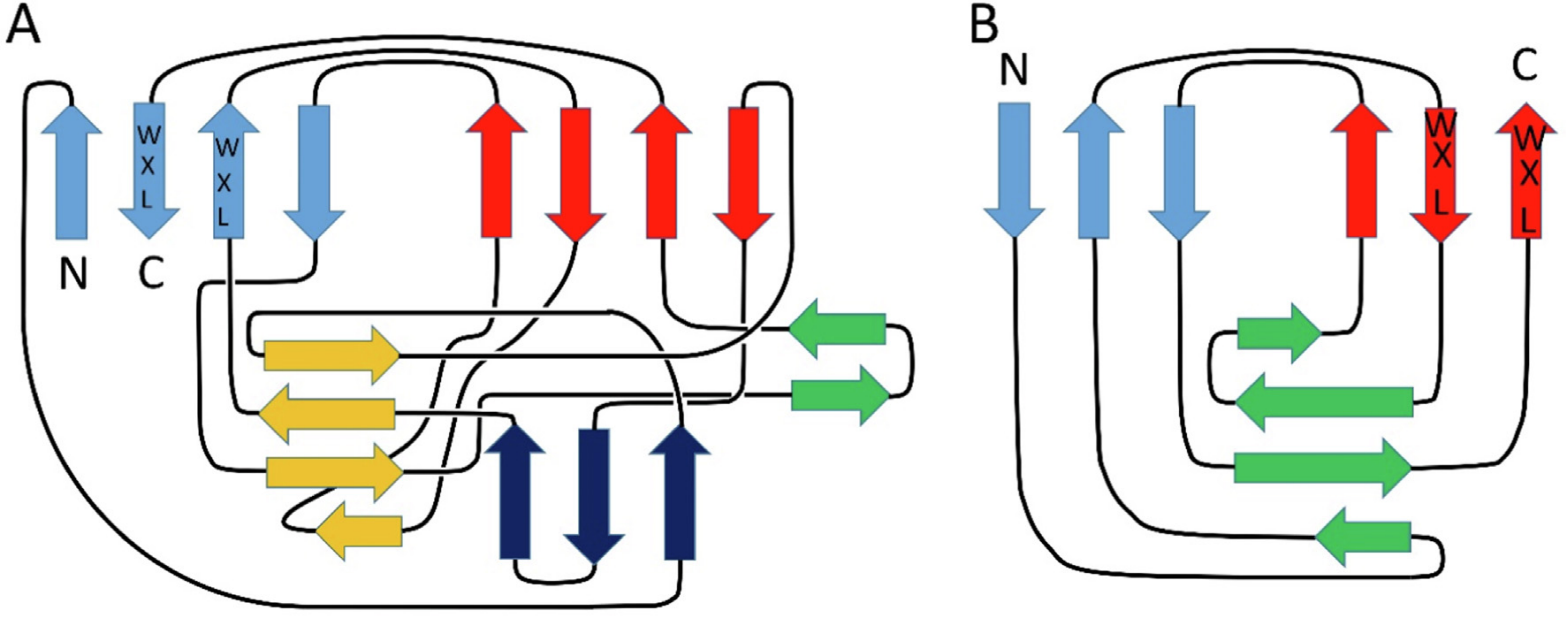
Secondary structure topology diagrams for (A) the small WxL domain SwpE2 and (B) the large WxL domain LwpA. The N- and C-termini are marked, as are the locations of the two WxL motifs, which are found on adjacent antiparallel strands.

The most interesting observation from these comparisons is that although the two WxL sequence motifs are well conserved across small and large WxL (Fig. 2), and the two domains are both composed largely of antiparallel β-sheets, in detail the structures of the two domains are completely different. This is most clearly apparent from topology diagrams of the regular secondary structure (Fig. 8). These show that the large WxL domain is smaller and simpler than the small WxL domain, and that there is little in common between them.

For both domains, the two WxL motifs are found on two adjacent β-strands. A detailed analysis of the structure predictions for the two domains demonstrates that they have more in common than just this sequence location, which provides clues as to the possible functions of the WxL motifs.

**Fig. 9:**
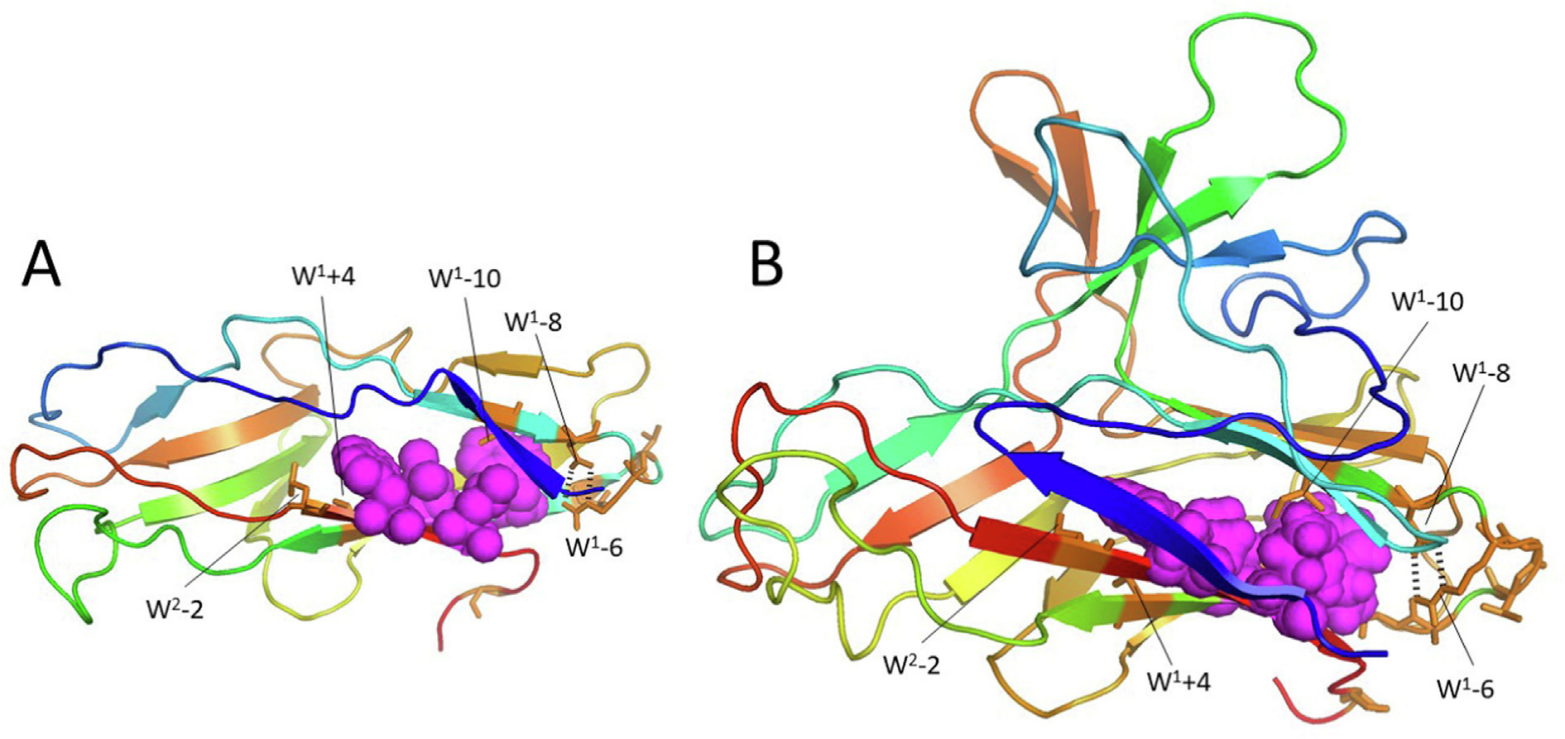
AlphaFold models for (A) the large WxL domain from LB3, (B) the small WxL domain from SwpE2. The chain is shown in rainbow view from *N*-terminus (blue) to C-terminus (red). The W and L residues of the two WxL motifs are shown in magenta spheres. A selection of the conserved residues from the two WxL motifs is indicated in orange, where for example W^1^ + 4 indicates the residue 4 residues after the tryptophan from the first WxL motif. A pair of hydrogen bonds between Asp (W^1^-8) and Arg (W^1^-6) is indicated by black dashes. For orientation with the topology diagram in Fig. 8, the sheet containing the WxL motifs is under the magenta spheres, and the sheet that forms a greek key pair with it (ie the red and cyan sheets from Fig. 8) is above the magenta spheres.

The three-dimensional AlphaFold models for the large WxL domain from LwpLB3 and small WxL domain from SwpE2 are shown in Fig. 9. The figure shows that the conserved WxL motifs also have a conserved tertiary structure. The pair of WxL sidechains form a flat hydrophobic platform, with the two leucines in the center and the two tryptophans forming large hydrophobic buttresses on each side. This is strengthened by conserved hydrophobic residues on the left at W^1^ + 4 and W^2^-2, and also by a hydrophobic residue at W^1^-10, which is contributed by the strand positioned above the WxL motif and further enlarges the hydrophobic core. The conserved residues Asp W^1^-8 and Arg W^1^-6 form a pair of hydrogen bonds between their sidechains that help to stabilise the start of a turn between the strand containing W^1^-10 and the first WxL strand, while the conserved GTxAG sequence following them forms the turn itself. Of particular interest is the *N*-terminal strand, shown in blue. This is in a similar place in both structures, despite the fact that in the topology diagrams it occupies quite different positions, being part of the upper β-sheet in LwpLB3, and part of the lower β-sheet in SwpE2. It is connected to the rest of the domain by a long sequence lacking regular secondary structure. We therefore hypothesise that the rationale for the conserved WxL motifs involves a structural rearrangement in this region, involving a rearrangement of the *N*-terminal strand, with the WxL platform forming a solid base for the structural change. The two pairs of WxL sidechains are buried in the structure and do not appear to play any role in ligand recognition or binding.

### 3.6 Ligand binding

The 3D Ligand site webserver was-michaelislab.org was used to identify potential ligands for the WxL domains (See Table S9). For small WxL, the significant ligands identified were only the metal ions calcium and copper. However, for large WxL, it identified *N*-acetyl glucosamine (NAG) as a likely ligand. NAG forms half of the peptidoglycan backbone, further confirming the likely role of the large WxL domain in binding to peptidoglycan. The binding site for NAG is at the back of the domain, behind the WxL motifs in the view shown in Fig. 9 (Fig. S4). This would imply that any structural changes involving the *N*-terminal strand are on a surface pointing away from the bacterial surface towards a potential host.

Docking was also conducted using the program MOE (Chemical Computing Group). This identified both NAG and NAM (*N*-acetyl muramic acid) as possible ligands for both small and large WxL, with the binding sites being at similar locations to that described above.

### 3.7 Protein-Protein interactions

The STRING webserver was used to find interaction partners of WxL proteins. The analysis concluded that WxL has a strong interaction with itself and with DUF916 proteins (Fig. S5). Galloway-Peña et al. also reported the same results, confirmed by biocore analysis, that DUFA protein (a DUF916 protein) showed self association and association with SwpA and LwpA protein (Galloway-Peña et al., 2015).

## 4 Discussion

The results presented here provide a coherent description of the structure and function of WxL domains. There are two different types of WxL domain, described as small and large. They are characterized by two conserved sequence motifs containing the sequence WxL, with a number of conserved residues in both motifs. WxL proteins are found almost exclusively in gut commensals, providing the first hint that they may be involved in attaching bacteria to their host’s endothelial layer. They occur in gene clusters, in which there is always one small WxL, one large WxL and one DUF916 domain. In addition there is often a protein containing the sequence LPxTG, which is used to attach the protein covalently to the peptidoglycan layer. The DUF916 is generally part of a pair with a DUF3324 domain, with often no other domains present in the predicted protein.

The small WxL domain is typically around 180 residues long, with no other domains present in the protein. By contrast, the large WxL domain is shorter, but is found as the C-terminal domain of much longer proteins that consist of a series of domains, many of which have been identified as adhesins. Large WxL proteins are predicted to consist of a roughly linear string of domains (Fig. 5), presumably extending out from the bacterial surface with the C-terminal WxL domain attached to the bacterial peptidoglycan layer and the other domains available for interaction with the host. Small and large WxL domains have a common pair of WxL motifs, which are located on adjacent β-strands and form a hydrophobic platform which is buried inside the protein, covered by the *N*-terminal strand. The WxL motifs therefore do not appear to be exposed on the surface and do not interact with binding partners. We have suggested that there may be a conformational change involving the *N*-terminal strand, but this is unlikely to be so large as to expose the WxL motifs on the surface. It is therefore concluded that the WxL motifs have a largely structural role, rather than being directly involved in host recognition or immune evasion.

These considerations lead to a model in which the proteins in the WxL gene cluster (minimally small WxL, large WxL and DUF916) assemble together, attached to peptidoglycan via small and large WxL domains. Small WxL protein contains no other domains and thus is presumably a core part of this complex. The other domains in the cluster (ie DUF3324 and other domains on large WxL) extend out, away from the bacterial surface, and are available for attachment to the host and also potentially for immune evasion.

## Declaration of Competing Interest

The authors declare that they have no known competing financial interests or personal relationships that could have appeared to influence the work reported in this paper.
