# Supplementary material for: Bioinformatic analysis of WxL domain proteins
Source: Saudi J Biol Sci. 2022 Dec 7;30(2):103526. doi: 10.1016/j.sjbs.2022.103526 (PMC9772566; doi:10.1016/j.sjbs.2022.103526)
Supplement: Supplementary data 1 [file mmc1.docx]

**Bioinformatic analysis of WxL domain proteins**

Mahreen Ul Hassan and Mike P. Williamson

**Supplementary data**

**Table S1. Characteristics of Large WxL and Small WxL proteins**


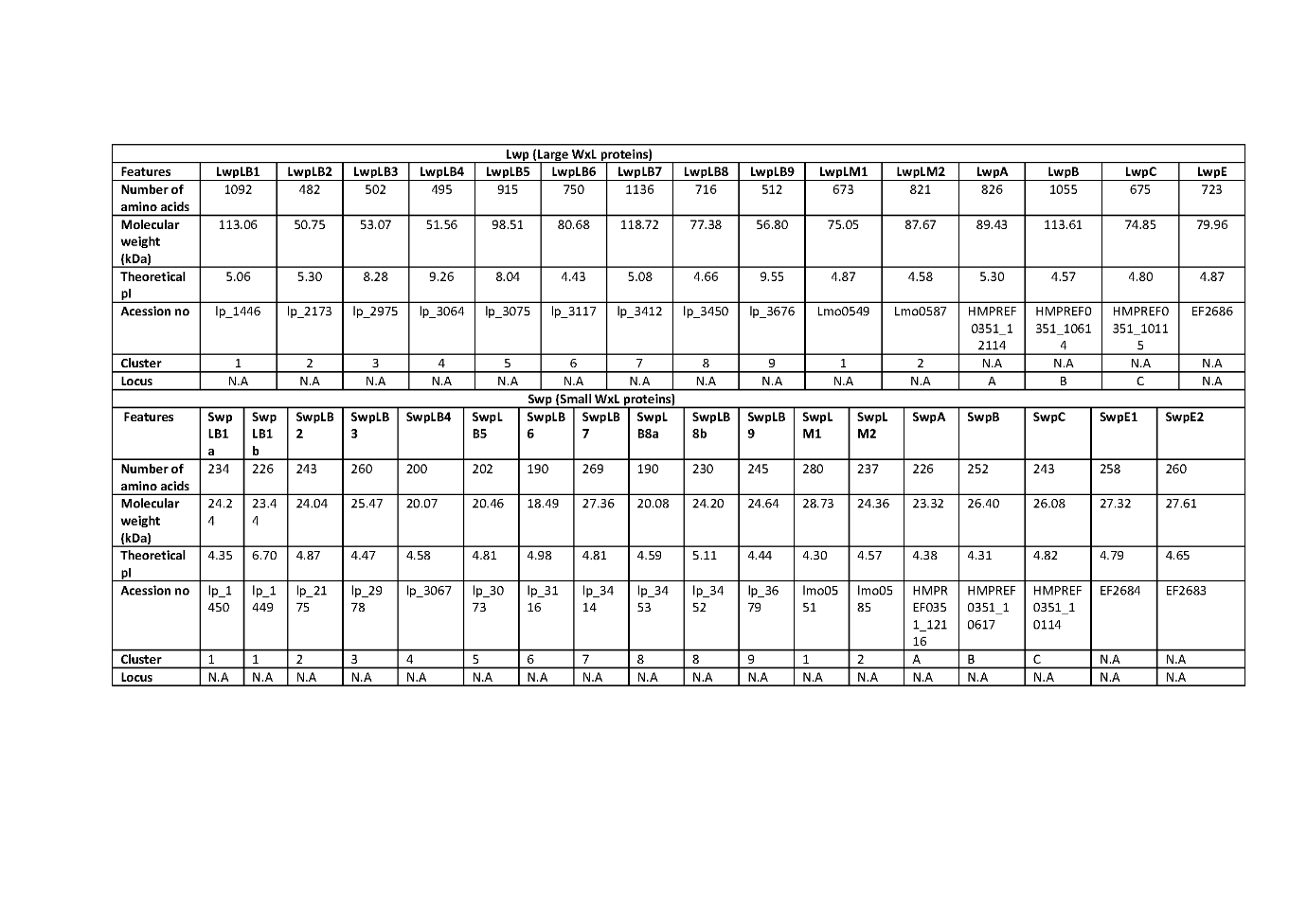


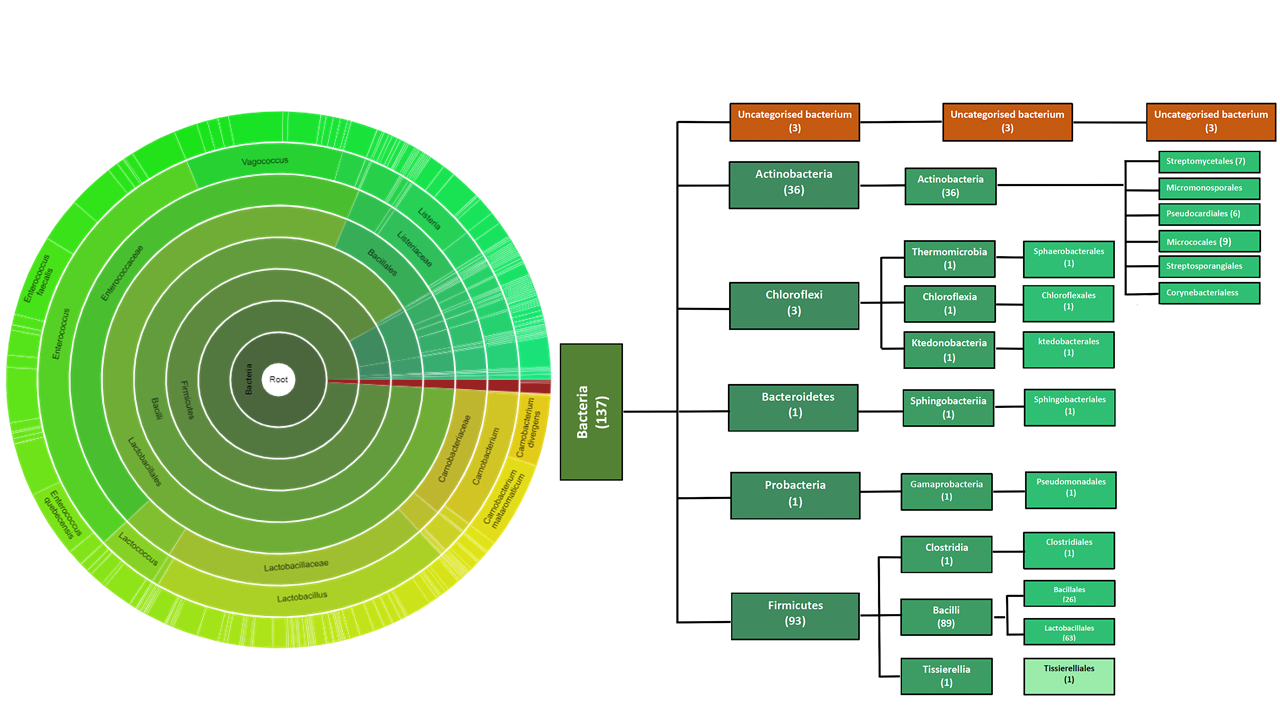


**Figure S1. Distribution of WxL domains among bacterial species**. The phylogenetic distribution of WxL domains is depicted in a sunburst chart, produced from the Pfam database (El-Gebali et al., 2009). The tree was constructed by considering the taxonomic lineage of each sequence that resembles this family and coloring it according to the assignment in Pfam.


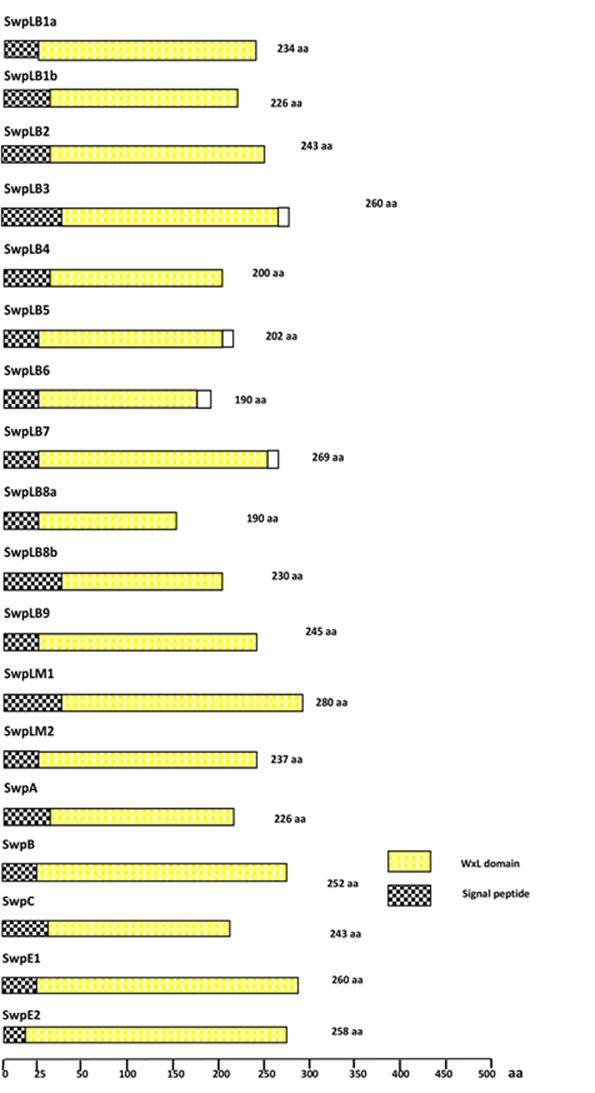


**Figure S2. Schematic representation of domain structure of Small WxL proteins.** SwpLB1a to SwpLB9 stand for Small WxL protein of clusters 1 to 9 of *L. plantarum* WCFS1; SwpLM1 and 2 stand for Small WxL protein on cluster 1 and 2 of *L. monocytogenes*; SwpA SwpB and SwpC stand for locus A, locus B and locus C of Small WxL protein of *E. faecium* DO; SwpE1 and SwpE2 are two small WxL proteins of *E. faecalis* V583.

**Table S2. Position of different domains within different Small WxL proteins detected by BlastP analysis and Motif Finder.**


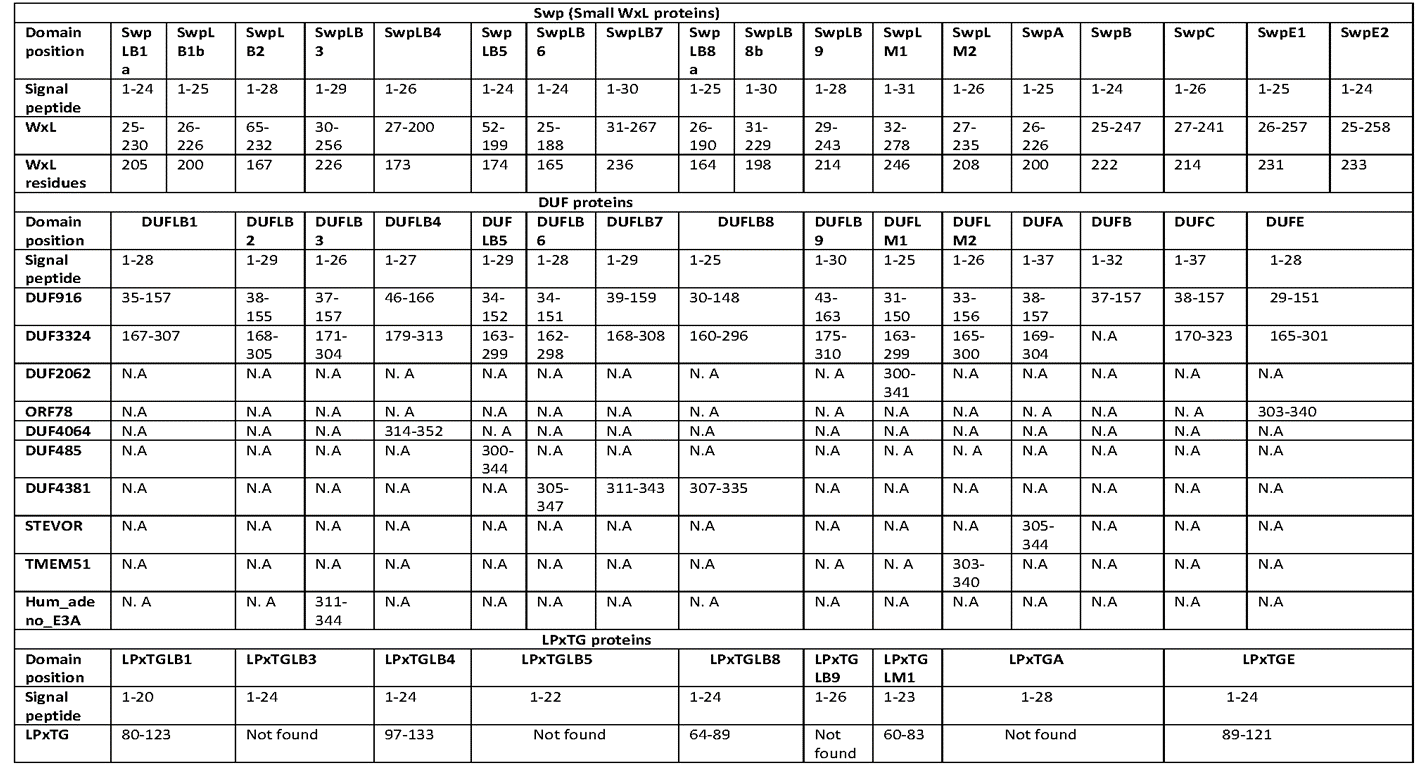


**Table S3. Position of different domains within different Large WxL proteins detected by BlastP analysis and Motif Finder.**


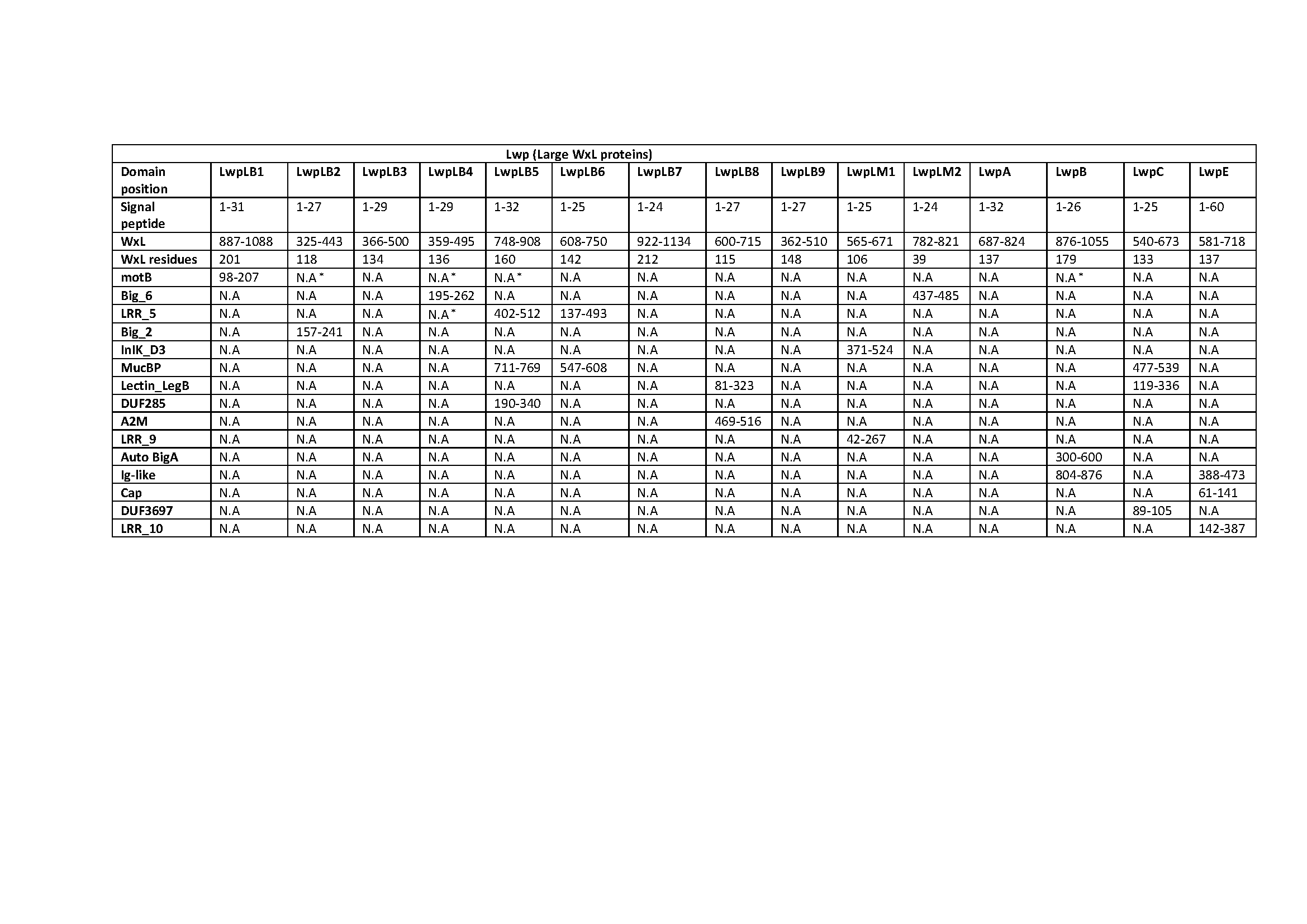


*N. A: not applicable.

**Table S4. Robetta analysis of large and small WxL predictions.**

| **Robetta analysis** | | | | | | | | | |
| --- | --- | --- | --- | --- | --- | --- | --- | --- | --- |
| **Features** | **LwpA-WxL** | **LwpB-WxL** | **LwpC-WxL** | **LwpE-WxL** | **SwpA** | **SwpB** | **SwpC** | **SwpE_1_** | **SwpE_2_** |
| **Structure confidence** | 81% | 63% | 88% | 53% | 74% | 71% | 76% | 73% | 77% |
| **Protein Coverage** | 100% | 100% | 100% | 100% | 100% | 100% | 100% | 100% | 100% |
| **Residues coverage** | 141 | 179 | 1-675 | 1-723 | 1--226 | 1-252 | 1-243 | 1-256 | 1-258 |
| **Modelling Method** | TrRefineRosetta | | | | | | | | |


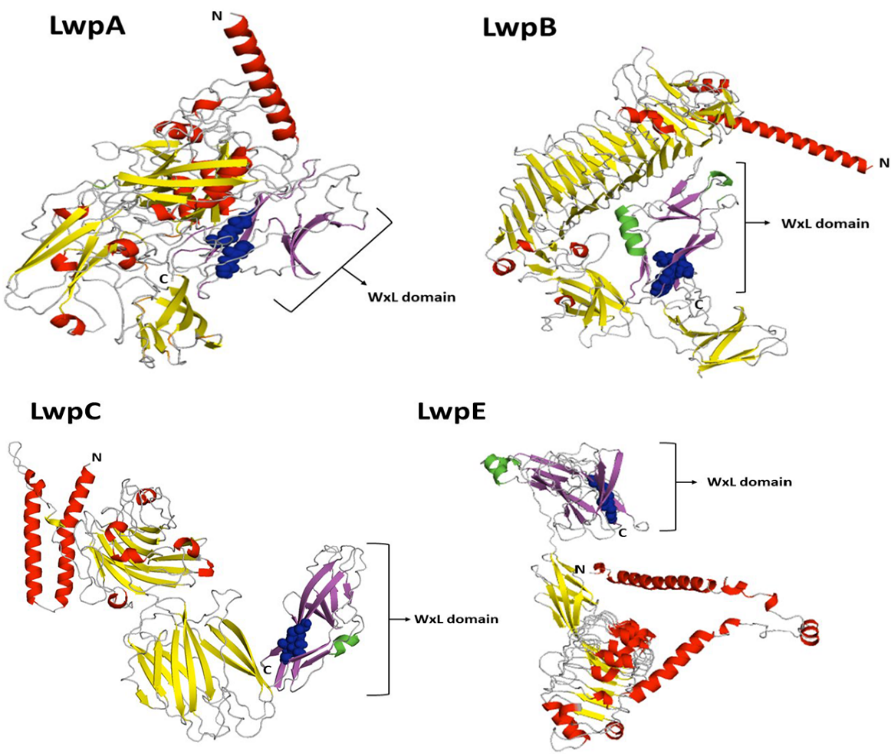


**Figure S3. Robetta analysis of Large WxL proteins.** WxL domain beta sheets are coloured in purple and alpha helix is coloured in green. The structures were predicted in April 2021. Blue spheres represent the two WxL motifs.

**Table S5. Evalution of Robetta models by PROCHECK, VERIFY 3D, ERRAT and PROVE**

| **Protein Name** | **Procheck** | | | | **Verify 3D** | **ERRAT** | **PROVE** |
| --- | --- | --- | --- | --- | --- | --- | --- |
|  | **Core** | **Allowed** | **Generously** | **Disallowed** | **3D-ID score** | **Quality factor** | **Z-score** |
| **SwpA** | 81.9% | 13.0% | 0.5% | 0.5% | 87.1% | 81.6% | 3.5 |
| **SwpB** | 81.3% | 14.5% | 2.8% | 1.4% | 78.5% | 84.5% | ERROR |
| **SwpC** | 86.7% | 12.8% | 0.5% | 0.0% | 81.0% | 86.7% | 1.0 |
| **SwpE1** | 86.5% | 12.1% | 0.9% | 0.4% | 83.3% | 93.5% | 0.43 |
| **SwpE2** | 86.8% | 12.3% | 0.0% | 0.9% | 78.0% | 87.0 % | Fails |
| **LwpA** | 79.0% | 20.1% | 0.7% | 0.1% | 87.2% | 83.9% | 0.8 Fails |
| **LwpB** | 83.8% | 14.5% | 1.2% | 0.6% | 91.46% | 87.7 % | 0.8 Fails |
| **LwpC** | 84.2% | 13.3% | 1.0% | 1.5% | 90.96% | 90.45% | 0.42(Fails) |
| **LwpE** | 78.3% | 19.8% | 0.6% | 1.2% | 72.8% | 89.2% | 0.2 |

**Table S6. Evaluation of AlphaFold models by PROCHECK, VERIFY 3D, ERRAT and PROVE**

| **Protein Name** | **Procheck** | | | | **Verify 3D** | **ERRAT** | **PROVE** |
| --- | --- | --- | --- | --- | --- | --- | --- |
|  | **Core** | **Allowed** | **Generously** | **Disallowed** | **3D-ID score** | **Quality factor** | **Z-score** |
| **SwpA** | 83.8% | 15.7% | 0.5% | 0.0% | 83.6% | 85.4% | 0.4 |
| **SwpB** | 84.1% | 10.3% | 3.3 % | 2.3 % | 80.9 % | 90.9 % | ERROR |
| **SwpC** | 86.7% | 12.8% | 0.5% | 0.0% | 81.0% | 92.17 % | 1.0 |
| **SwpE1** | 87.3% | 11.8% | 0.5% | 0.5% | 81.1% | 96.7% | 2.4 |
| **SwpE2** | 88.8% | 9.0% | 1.3% | 0.9% | 76.36% | 97.1 % | 1.4 Fails |
| **LwpA** | 92.4% | 7.6% | 0.0% | 0.0% | 100% | 92.24% | 0.8 Fails |
| **LwpB** | 83.8% | 14.5% | 1.2% | 0.6% | 91.46% | 87.7 % | 0.8 Fails |
| **LwpC** | 84.2% | 13.3% | 1.0% | 1.5% | 90.96% | 90.45% | 0.42(Fails) |
| **LwpE** | 92.5% | 7.1% | 0.0% | 0.4% | 98.0% | 97.7% | 0.5 |

**Table S7. Ramachandran distributions of Robetta 3D models of WxL proteins**

| **Protein** | **Most favoured region** | **Additional allowed region** | **Generously allowed region** | **Disallowed region** | **Non-glycine and non-proline residues** | **End-residues (excl. Gly and Pro)** | **Glycine residues** | **Proline residues** |
| --- | --- | --- | --- | --- | --- | --- | --- | --- |
| **SwpA**  **(226)** | 155  (85.9%) | 23  (13.0%) | 1  (0.5%) | 1  (0.5%) | 185  (100.0%) | 02 | 22 | 17 |
| **SwpB**  **(252)** | 174  (81.3%) | 31  (14.5 %) | 6  (2.8 %) | 3  (1.4 %) | 214  (100.0%) | 02 | 21 | 15 |
| **SwpC**  **(243)** | 176  (86.7%) | 26  (12.8 %) | 1  (0.5 %) | 0  (0.0 %) | 203  (100.0%) | 02 | 20 | 18 |
| **SwpE1**  **(258)** | 193  (86.5%) | 27  (12.5 %) | 2  (0.9 %) | 1  (0.4 %) | 223  (100.0%) | 02 | 18 | 15 |
| **SwpE2**  **(260)** | 191  (86.8%) | 27  (12.3 %) | 0  (0.0 %) | 2  (0.9 %) | 220  (100.0%) | 02 | 18 | 20 |
| **LwpA**  **(826)** | 569  (79.0%) | 145  (20.1 %) | 5  (0.7 %) | 1  (0.1 %) | 720  (100.0%) | 02 | 64 | 40 |
| **LwpB**  **(826)** | 713  (83.8%) | 145  (20.1 %) | 5  (0.7 %) | 1  (0.1 %) | 720  (100.0%) | 02 | 64 | 40 |
| **LwpC**  **(675)** | 502  (84.2%) | 79  (13.3 %) | 6  (1.0 %) | 9  (1.5 %) | 596  (100.0%) | 02 | 53 | 24 |
| **LwpE**  **(723)** | 513  (78.3%) | 130  (19.8 %) | 4  (0.6 %) | 8  (1.2 %) | 655  (100.0%) | 02 | 32 | 34 |

**Table S8. Ramachandran plot calculations of 3D model of WxL proteins produced by AlphaFold**

| **Protein** | **Most favoured region** | **Additional allowed region** | **Generously allowed region** | **Disallowed region** | **Non-glycine and non-proline residues** | **End-residues (excl. Gly and Pro)** | **Glycine residues** | **Proline residues** |
| --- | --- | --- | --- | --- | --- | --- | --- | --- |
| **SwpA**  **(226)** | 155  (83.8%) | 29  (15.7%) | 1  (0.5%) | 0  (0.0%) | 185  (100.0%) | 02 | 22 | 17 |
| **SwpB**  **(252)** | 180  (84.1 %) | 22  (10.3 %) | 7  (2.8 %) | 5  (2.3 %) | 214  (100.0%) | 02 | 21 | 15 |
| **SwpC**  **(243)** | 181  (89.2%) | 22  (10.8 %) | 0  (0.0 %) | 0  (0.0 %) | 203  (100.0%) | 02 | 20 | 18 |
| **SwpE1**  **(258)** | 192  (87.3%) | 26  (11.8 %) | 1  (0.5 %) | 1  (0.5 %) | 220  (100.0%) | 02 | 18 | 20 |
| **SwpE2**  **(260)** | 198  (88.8%) | 20  (9.0 %) | 3.0  (1.3 %) | 2  (0.9 %) | 223  (100.0%) | 02 | 18 | 15 |
| **LwpA**  **(826)** | 556  (92.4%) | 9  (7.6 %) | 5  (0.7 %) | 1  (0.1 %) | 118  (100.0%) | 02 | 13 | 8 |
| **LwpB**  **(826)** | 713  (83.8%) | 145  (20.1 %) | 5  (0.7 %) | 1  (0.1 %) | 720  (100.0%) | 02 | 64 | 40 |
| **LwpC**  **(675)** | 502  (89.2%) | 79  (15.3 %) | 6  (1.0 %) | 9  (1.5 %) | 596  (100.0%) | 02 | 53 | 24 |
| **LwpE**  **(723)** | 513  (92.5%) | 16  (7.1 %) | 0  (0.0 %) | 1  (0.4 %) | 655  (100.0%) | 02 | 32 | 34 |

**Table S9. 3D-ligand prediction of the binding site**

| **Protein** | **Predicted binding site** | **Heterogen present in predicted binding site** |
| --- | --- | --- |
| **SwpA**  **(226)** | ASN^48^,PRO^49^,THR^50^,AS^P51^,PRO^52^,THR^148^,  THR^150^,LYS^152^,LEU^192^ and VAL^194^ | BMA (Butyl Methacrylate) |
| **SwpB**  **(252)** | LEU^85^,PHE^87^,ALA^100^,PHE^157^,LEU^210^,SER^220^  VAL^221^ and GLU^222^ | CU (copper) |
| **SwpC**  **(243)** | GLU^33^ | CA (calcium) |
| **SwpE1**  **(258)** | VAL^36^,PHE^94^ | CA (calcium) |
| **SwpE2**  **(260)** | LYS^101^,LYS^166^ and THR^232^ | CU (copper) |
| **LwpA**  **(826)** | THR^51^,ASN^87^,THR^140^ and SER^141^ | NAG7 |
| **LwpB**  **(826)** | LYS^47^ | NAG1 |
| **LwpC**  **(675)** | TRP^46^ ,PHE^86^ ,GLU^87^ ,SER^94^ ,ASP^95^ ,  LEU^96^ ,SER^97^  and SER^98^ | NAG1 |
| **LwpE**  **(723)** | HIS^90^ ,ASN^91^ ,ALA^93^ ,THR^99^ ,ASN^100^ ,  ILE^101^ ,ASP^103^ and GLN^104^ | NAG4 |


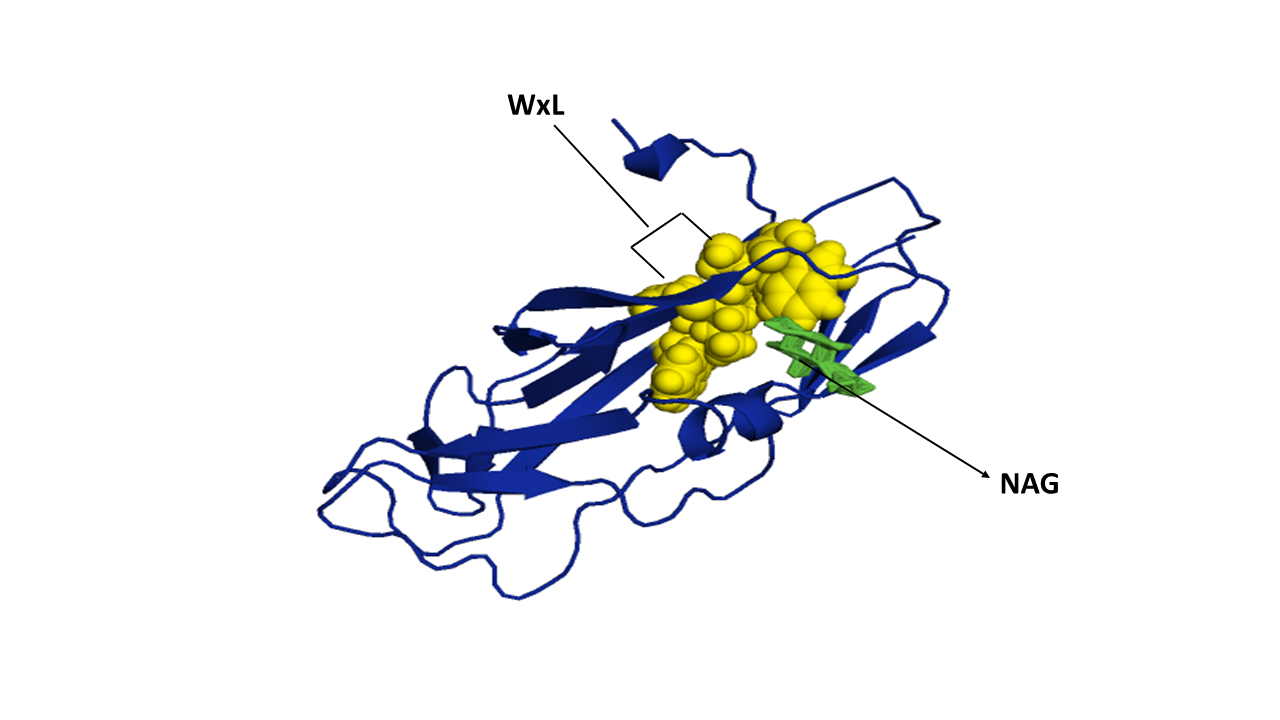


**Figure S4. LwpE interaction with NAG4 , conducted using 3-D ligand.**

**
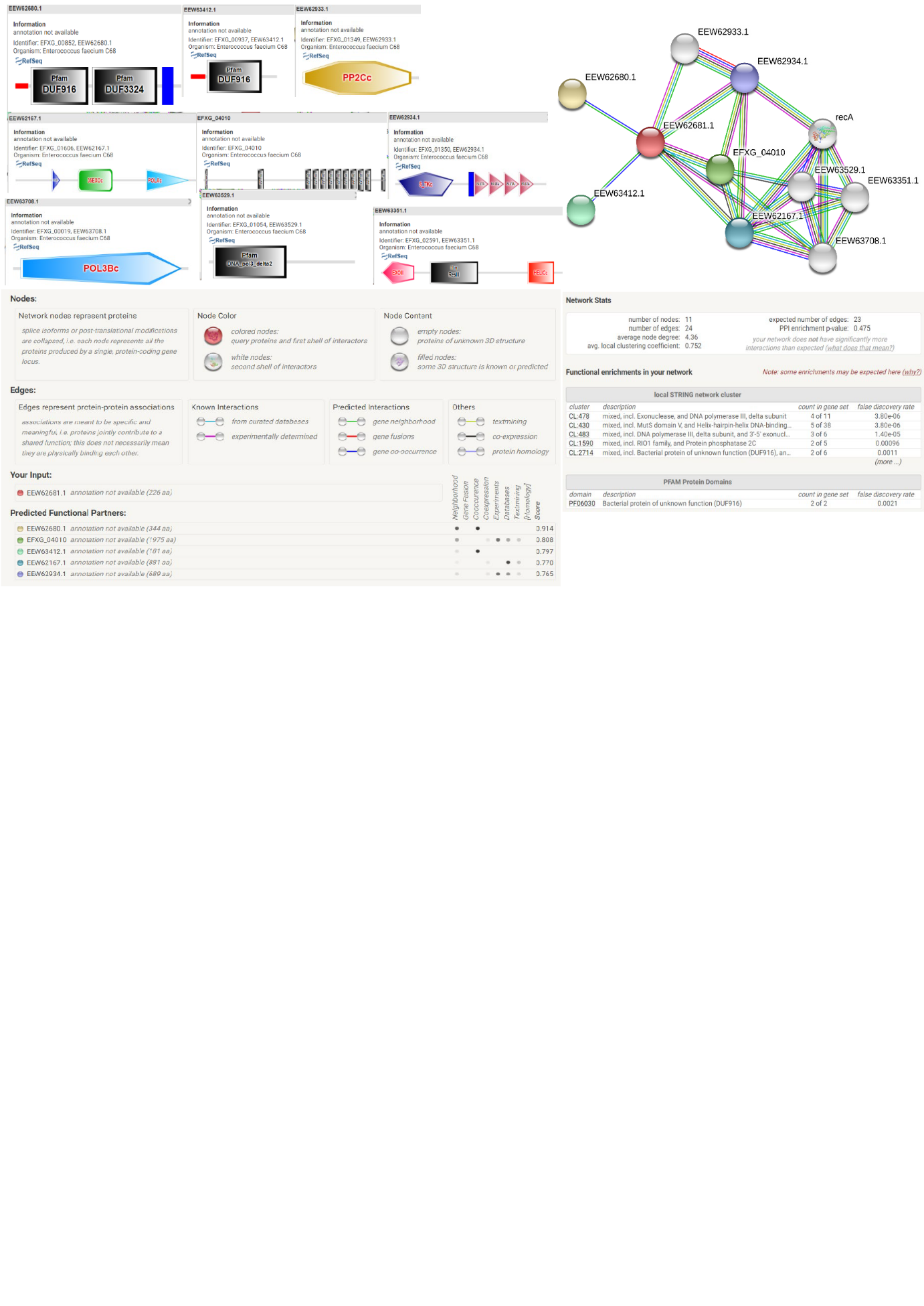
**

**Figure S5. SwpA interaction with other proteins, conducted using STRING.**
